# Methylation status of *DDIT3 *gene in Chronic Myeloid Leukemia

**DOI:** 10.1186/1756-9966-29-54

**Published:** 2010-05-23

**Authors:** Ya-li Wang, Jun Qian, Jiang Lin, Dong-ming Yao, Zhen Qian, Zhao-hui Zhu, Jian-yong Li

**Affiliations:** 1 Department of Hematology, Affiliated People's Hospital of Jiangsu University, Zhenjiang, Jiangsu, 212002, PR China; 2 Department of Hematology, The First Hospital of Nanjing Medical University, Jiangsu Provincial People's Hospital, Nanjing, Jiangsu, 210029, PR China

## Abstract

**Background:**

DNA-damage-inducible transcript 3 (*DDIT3*), a candidate tumor suppressor gene (TSG), has been found involved in the regulation of cellular growth and differentiation. The epigenetic changes of TSGs are recently recognized as an abnormal mechanism contributing to the development of chronic myeloid leukemia (CML). The aim of this study was to investigate the methylation status of *DDIT3 *gene in CML patients.

**Methods:**

The methylation status of *DDIT3 *promoter was detected in the bone marrow mononuclear cells from 53 patients with CML using methylation-specific PCR (MSP). The expression levels of *DDIT3 *and *bcr/abl *transcript were determined by real-time quantitative PCR (RQ-PCR). Clinical data of these patients were collected and analyzed.

**Results:**

The aberrant methylation of *DDIT3 *gene promoter was found in 35 of 53 (66%) CML cases. Correlation was not found between *DDIT3 *promoter hypermethylation and the age, sex, hemoglobin concentration, platelet counts, chromosomal abnormalities, *bcr/abl *transcript, and staging of CML patients (*P *> 0.05), but found between *DDIT3 *promoter hypermethylation and WBC counts of CML cases (R = 0.781, *P *< 0.001). The level of *DDIT3 *transcript in CML patients was significantly lower than that in controls (median 3.28 vs 19.69, *P *< 0.001), however, there was no difference in the level of *DDIT3 *transcript between methylation-positive CML cases (0.05-65.32, median 2.13) and methylation- negative CML cases (0.12-126.04, median 3.92) (*P *> 0.05).

**Conclusion:**

Our results demonstrate that aberrant methylation of *DDIT3 *occurs in CML frequently.

## Background

Chronic myeloid leukemia (CML) is a stem cell disease characterized by excessive accumulation of clonal myeloid cells in hematopoietic tissues. Almost all patients with CML present the common cytogenetic abnormality of the t(9;22) and the *bcr/abl *fusion gene which is generated by the translocation. Clinically CML can be divided into three phases: the chronic phase (CP), the accelerated phase (AP), the blast crisis (BC) [1,2]. BC is the last stage of CML disease progress, in which hematopoietic differentiation become arrested and immature blasts accumulate in the bone marrow and spill into the circulation. The mechanisms responsible for transition of CP into BC remain poorly understood [3].

In the pathogenesis of leukemias and other cancers, gene silencing by aberrant DNA methylation is a frequent event [4,5]. The methylation of several tumor suppressor genes (TSGs) including E-cadherin, death-associated protein kinase (*DAPK*), estrogen receptor (*ER*), and the cell cycle regulating genes (*P15*^*INK4B *^and *P16*^*INK4A*^), has been confirmed associated with the development and progression of CML [6-9].

DNA-damage-inducible transcript 3 (*DDIT3*), also named CCAAT/enhancer binding protein zeta (*C/EBPζ*), is expressed ubiquitously and can be induced by a wide variety of treatments such as DNA lesion, hypoglycaemia, radiation and cellular stress. Several studies have confirmed the role of *DDIT3 *in the regulation of cellular growth and differentiation [10-13]. The overexpression of *DDIT3 *transcript has been found to induce increased apoptosis of myeloid cells and block cells in the progression from G1 to S phase [14,15]. The level of *DDIT3 *transcript has been revealed down-regulated in myeloid malignancies in our previous study [16]. The other five members of C/EBP proteins also play important roles in cellular proliferation and terminal differentiation of hematopoietic cells. Recently, two members of *C/EBP *family, *C/EBPα *and *C/EBPδ*, have been found to be silenced by aberrant methylation in acute myeloid leukemia (AML) [17-19]. However, the methylation status of *DDIT3 *promoter has not yet been studied in leukemia. The primary aim of this study is to investigate the methylation status of *DDIT3 *promoter in CML patients and determine the association of *DDIT3 *methylation with the patients' clinical features.

## Materials and methods

### Patients and samples

Fifty-three cases of CML seen at the Affiliated People's Hospital of Jiangsu University were selected for this study based on the availability of stored leukemic cells. Thirty-five patients were in CP, three in AP, and fifteen in BC. The diagnosis of CP, AP and BC was established according to conventional criteria. Briefly, CP was defined as having within the peripheral blood and bone marrow less than 10% blasts, less than 20% basophils, and less than 30% blasts plus promyelocytes, with t(9:22) translocation or *bcr/abl *transcript. AP was defined as having blasts ≥ 10%, blasts and promyelocytes ≥ 30%, basophils ≥ 20%, platelets ≤100 × 10^9^/L unrelated to therapy, or cytogenetic clonal evolution. BC was defined as the presence of ≥ 20% peripheral or bone marrow (BM) blasts, or extramedullary blastic disease. The BM samples from all patients were harvested at the time of diagnosis and BM mononuclear cells (BMNCs) were isolated using Ficoll solution and washed twice in PBS and then frozen in -80°C. BM samples collected from thirteen donors of BM transplantation were used as controls. Placenta tissue of one healthy pregnant woman was used as the sample to prepare positive controls of methylated and unmethylated DNA. Informed consents were provided according to the Declaration of Helsinki.

### RNA isolation and Real-time quantitative PCR (RQ-PCR)

Total RNA was extracted from the BMNCs of CML patients by the guanidinium thiocyanate/acid phenol method using Trizol reagent (Invitrogen Life Technologies, USA) in accordance with the manufacturer's standard method. 2 μg of total RNA was reverse transcribed into cDNA by using random primers, 200 U of MMLV reverse transcriptase (InVitrogen), 0.5 mM dNTPs, 10 mM dithiothreitol, and 25 U of RNase inhibitor (InVitrogen). 40-μL RT reaction was performed at 37°C for 60 min, then at 95°C for 5 min. cDNA was stored in -20°C until assayed. *DDIT3 *and *bcr/abl *transcripts were quantified using RQ-PCR established previously [7,16].

### DNA isolation and bisulfite modification

DNA was isolated from BMNCs using Genomic DNA Purification Kit (Gentra, Minneapolis, MN, USA). 1 μg of genomic DNA was modified as described in manufacture's instruction using the CpGenome™ DNA Modification Kit (Chemicon, Ternecula, Canda). Modified DNA was resuspended in water and used immediately or stored at -80°C until used.

### Methylation-specific polymerase chain reaction (MSP)

DNA methylation status in the CpG island of *DDIT3 *promoter was determined by the MSP procedure described previously [20]. Primer sequences for the methylated (M) MSP reaction were 5'-GGTTCGATATTACGTCGATTTTTTAGC-3' (forward) and 5'-GCCGACATT AACCCCG-3' (reverse), and primer sequences for the unmethylated (U) MSP reaction were 5'-ATTTTTGGGTTTGATATTATGTTGATTTTTTAGTG-3' (forward) and 5'-CAAAAAA TAACACACCAACATTAACCCCA-3' (reverse). 25 μl of reaction mixture contained 1 × PCR buffer (containing 15 mmol/L MgCl_2_), 2.5 mmol/L dNTPs, 0.4 pmol/L primers, 1 U Hot start DNA polymerase (Takara, Tokyo, Japan), and 2 μl of modified DNA. PCR conditions were 95°C for 5 min, 35 cycles for 30 s at 94°C, 30 s at 63.1°C (M) or 62°C (U), 30 s at 72°C followed by a final 7 min extension step at 72°C. Amplification was carried out in a 9600 Perkin-Elmer Thermal Cycler (PerkinElmer Inc., Ramsey, MN, USA). Placenta DNA treated in vitro with SssI methyltransferase (New England Biolabs, Beverly, MA, USA) and untreated were used as positive controls for methylated and unmethylated templates, respectively. Negative control samples without DNA were included for each set of PCR. PCR products were analyzed on 3% agarose gels and visualized under UV illumination. To verify successful bisulfite modification of the DNA, a sequence containing cytosines would not be amplified after bisulfite modification by using primers that would only give an amplified product if the cytosines in the template sequence were not converted to uracils. For this purpose, a region of the β-actin promoter was amplified with every modified DNA sample. Samples that did not give a band were considered completely modified and further used for MSP analysis. Those cases were defined as methylation positive if their sample showed a visual band amplified with methylated-specific primers, even if the band was faint. All positive results were confirmed in at least two MSP analyses. Using 80 ng totally methylated placenta DNA serially diluted by totally unmethylated DNA, we determined that the sensitivity for a clear detection of a methylated allele was 2%. Positive products of M and U reaction from one patient in CP were cloned and sequenced (Shanghai GeneCore BioTechnologies Co., Ltd., Shanghai, China).

### Conventional cytogenetic analysis

Conventional cytogenetic investigation was carried out for these patients. Chromosomes were prepared routinely by the direct method or short-term culture of BM cells. Karyotypes were analyzed on R-banded metaphases. Chromosomal abnormalities were described according to the International System for Human Cytogenetic Nomenclature. CML patients were classified as three subgroups according to the types of cytogenetic abnormalities: t(9;22), variant t(9;22), and t(9;22) with additional alteration.

### Statistical analysis

Statistical analysis was performed using the SPSS 13.0 software package (SPSS, Chicago, IL, USA). Chi-square analysis and Fisher exact test were carried out to compare the difference of frequencies between groups of patients. Mann-Whitney's U-test was carried out to the difference of age, level of *DDIT3 *and *bcr/abl *transcript between the methylated and the unmethylated groups. The correlation between the frequency of *DDIT3 *promoter methylation and the clinical and hematologic parameters was analyzed with Spearman's rank correlation. For all analyses, the *P*-values were two-tailed, and a *P*-value of < 0.05 was considered statistically significant.

## Results and discussion

**Figure 1:**
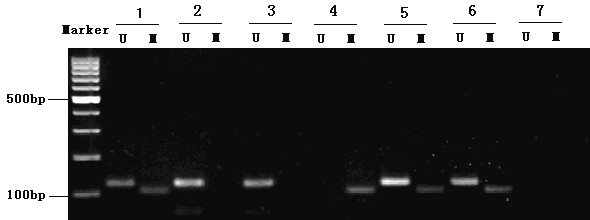
**MSP results of *DDIT3 *gene in CML**. U and M represent PCR results by using primer sets for methylated and unmethylated *DDIT3 *gene, respectively. 1: positive control (positive controls of methylation and unmethylation are genomic DNA of placenta which is modified with or without M.SssI); 2: sample of one BM donor; 3,4: samples of two cases at CP; 5: sample of one case at AP; 6: sample of one case at BC; 7: ddH2O; Mark: Gene Ruler™ 100 bp DNA Ladder.

**Figure 2:**
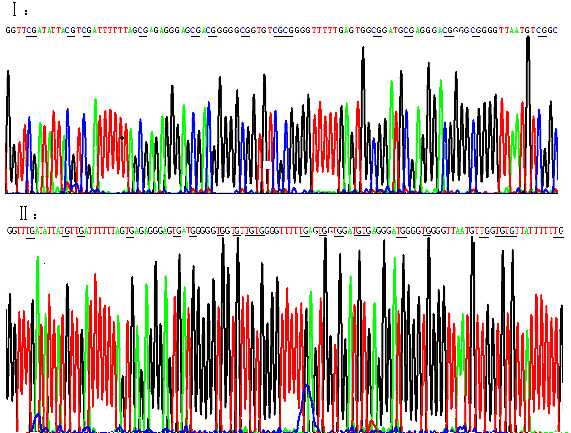
**The sequencing results of MSP products in one patient with CML**. I: The sequencing result of methylated product, CG was not changed after bisulfite treatment; II: The sequencing result of unmethylated product, T was replaced by C after bisulfite treatment.

Thirty-five patients (66%) showed *DDIT3 *hypermethylation that was not found in all controls (*P *< 0.001). The representative results of MSP were shown in Fig 1. The sequencing of PCR products from one CML patient confirmed the MSP results, shown in Fig 2. There were no significant correlations between the methylation status of *DDIT3 *promoter and the clinical features, such as age, sex, initial hemoglobin level, platelet counts, chromosomal abnormalities, and *bcr/abl *transcript (*P *> 0.05). The level of *DDIT3 *transcripts in CML patients (0.05-126.04, median 3.28) was significantly lower than that in controls (6.19-82.16, median 22.37) (*P *< 0.001). Although methylation-positive CML cases had lower *DDIT3 *transcript level than those methylation-negative cases, however, the difference was not significant (Table 1). This result may be associated with the low number of patients studied. Other mechanisms besides DNA methylation might be also involved in the regulation of *DDIT3 *expression. More cases should be further studied to determine the impact of *DDIT3 *methylation on the regulation of transcription.

**Table 1:** Correlation between methylation of *DDIT3 *gene and the clinical characteristics of CML patients.

	Status of *DDIT3 *methylation			
	
Patient's parameters	Patients with methylated *DDIT3 * (n = 35)	Patients with unmethelated *DDIT3 * (n = 18)	Total (n = 53)	*P *value
Ages (yr) ^1^	48 (21-73)	40 (17-83)	45 (17-83)	0.225
Sex (male/female)	28/7	10/8	38/15	0.106
WBC (×10^9^/L) ^1^	38.0 (2.2-178.6)	161.8 (4.1-235.2)	75.6 (2.2-235.2)	0.007
Hemoglobin (g/dL)^1^	9.9 (4.9-14.8)	9.3 (5.2-14.3)	9.5 (4.9-14.8)	0.963
Plateletcounts (×10^9^/L) ^1^	264 (20-1494)	263 (24-870)	264 (20-1494)	0.844
Cytogenetics				0.542
t(9;22)	26 (63%)	15 (37%)	41	
variant t(9;22)	2 (100%)	0 (0%)	2	
t(9;22) with additional alteration	7 (70%)	3 (30%)	10	
Staging				0.256
CP	24 (68%)	11 (32%)	35	
AP	3 (100%)	0 (0%)	3	
BC	8 (53%)	7 (47%)	15	
*bcr/abl *transcript	4.82 (0.28-877.94)	3.37 (0.26-221.77)	3.96 (0.26-877.94)	0.583
*DDIT3 *transcript	2.13 (0.05-65.32)	3.92 (0.12-126.04)	3.28 (0.05-126.04)	0.152

WBC, white blood cells; CP, chronic phase; AP, accelerated phase; BC, blast crisis. ^1 ^Median (range).

The correlation was found between *DDIT3 *promoter hypermethylation and white blood cells (WBC) (R = -0.781, *P *< 0.001). Using partial correlation analysis to exclude the effect of staging, *DDIT3 *promoter hypermethylation was also correlated with WBC (R = -0.474, *P *= 0.001). WBC of patients with methylation was significantly lower than that of patients without methylation (Table 1). We postulate that the down-regulation of *DDIT3 *transcripts caused by promoter methylation fails to induce mitotic cessation of injured cells, which eventually results in the delivery of DNA lesions to offspring cells and the susceptibility to carcinogenesis. However, the offspring cells gaining *DDIT3 *methylation might be prone to apoptosis or growth inhibition owing to other mechanisms.

The frequencies of *DDIT3 *promoter hypermethylation in CML patients in CP, AP and BC were shown in Table 1. However, correlation was not found between the frequency of *DDIT3 *promoter hypermethylation and different CML stages (*P *> 0.05). Our results suggested that the methylation of *DDIT3 *promoter might occur in the early stage of CML development. Further study on a more number of CML patients is needed to explore the role of *DDIT3 *methylation in the progression of CML.

*C/EBP *genes are believed to be critically involved in hematopoietic differentiation and leukemogenesis. Especially, the crucial role of *C/EBPα *in lineage determination during normal hematopoiesis is well established. *C/EBPα *mutations, contributing as an early event to leukemogenesis by inhibiting myeloid differentiation, are found in 10-15% of AML cases [19]. Recently, hypermethylation of *C/EBPα *promoter has also been identified in 12-51% of AML cases [18,19]. The systematic analysis has revealed that *C/EBPα *mutations or hypermethylation are associated with favorable karyotype or prognosis [18,19]. Hypermethylation of another C/EBP member, *C/EBPδ*, has been revealed in 35% AML patients [17]. These studies indicate that epigenetic alterations of *C/EBP *genes are involved in leukemia and can be used for disease stratification as well as therapeutic targets.

In conclusion, we demonstrate that aberrant methylation in the CpG island of the promoter region of *DDIT3 *gene is a common event in CML. However, further study will be needed to determine the role of *DDIT3 *methylation in the development, progress, and prognosis of CML.

## Competing interests

The authors declare that they have no competing interests.

## Authors' contributions

QJ and LJY designed the study, analyzed the data and wrote the manuscript; WY, LJ and YDM performed all experiments; QZ and ZZH gave assistance with technical performance and contributed to the writing of the manuscript.

## References

[B1] Quintás-Cardama A Cortes JE Chronic myeloid leukemia: diagnosis and treatment Mayo Clin Proc 2006 81 973 988 10.4065/81.7.973 16835977

[B2] Melo JV Barnes DJ Chronic myeloid leukaemia as a model of disease evolution in human cancer Nat Rev Cancer 2007 7 441 453 10.1038/nrc2147 17522713

[B3] Calabretta B Perrotti D The biology of CML blast crisis Blood 2004 103 4010 4022 10.1182/blood-2003-12-4111 14982876

[B4] Baylin SB Herman JG DNA hypermethylation in tumorigenesis: epigenetics joins genetics Trends Genet 2000 16 168 174 10.1016/S0168-9525(99)01971-X 10729832

[B5] Esteller M Aberrant DNA methylation as a cancer-inducing mechanism Annu Rev Pharmacol Toxicol 2005 45 629 656 10.1146/annurev.pharmtox.45.120403.095832 15822191

[B6] Issa JP Zehnbauer BA Civin CI Collector MI Sharkis SJ Davidson NE Kaufmann SH Baylin SB The estrogen receptor CpG island is methylated in most hematopoietic neoplasms Cancer Res 1996 56 973 977 8640788

[B7] Qian J Wang YL Lin J Yao DM Xu WR Wu CY Aberrant methylation of the death-associated protein kinase 1 (DAPK1) CpG inland in chronic myeloid leukemia Eur J Haematol 2009 82 119 123 10.1111/j.1600-0609.2008.01178.x 19018866

[B8] Melki JR Vincent PC Brown RD Clark SJ Hypermethyation of E-cadherin in leukemia Blood 2000 95 3208 3213 10807790

[B9] Herman JG Civin CI Issa JP Collector MI Skarkis SJ Baylin SB Distinct patterns of inactivation of p15INK4B and p16INK4A characterize the major types of hematological malignancies Cancer Res 1997 57 837 841 9041182

[B10] Maytin EV Habener JF Transcription factors C/EBP alpha, C/EBP beta, and CHOP (Gadd153) expressed during the differentiation program of keratinocytes in vitro and in vivo J Invest Dermatol 1998 110 238 246 10.1046/j.1523-1747.1998.00123.x 9506442

[B11] Tang QQ Lane MD Role of C/EBP homologous protein (CHOP-10) in the programmed activation of CCAAT/enhancer-binding protein-beta during adipogenesis Proc Natl Acad Sci USA 2000 97 12446 12450 10.1073/pnas.220425597 11050169 PMC18783

[B12] Pereira RC Delany AM Canalis E CCAAT/enhancer binding protein homologous protein (DDIT3) induces osteoblastic cell differentiation Endocrinology 2004 145 1952 1960 10.1210/en.2003-0868 14684614

[B13] Coutts M Cui K Davis KL Keutzer JC Sytkowski AJ Regulated expression and functional role of the transcription factor CHOP (GADD153) in erythroid growth and differentiation Blood 1999 93 3369 3378 10233889

[B14] Friedman AD GADD153/CHOP, a DNA damage-inducible protein, reduced CAAT/enhancer binding protein activities and increased apoptosis in 32D c13 myeloid cells Cancer Res 1996 56 3250 3256 8764117

[B15] Matsumoto M Minami M Takeda K Sakao Y Akira S Ectopic expression of CHOP (GADD153) induces apoptosis in M1 myeloblastic leukemia cells FEBS Lett 1996 395 143 147 10.1016/0014-5793(96)01016-2 8898082

[B16] Qian J Chen Z Lin J Wang W Cen J Decreased expression of CCAAT/enhancer binding protein zeta (C/EBPzeta) in patients with different myeloid diseases Leuk Res 2005 29 1435 1441 10.1016/j.leukres.2005.05.020 16005964

[B17] Agrawal S Hofmann WK Tidow N Ehrich M Boom D van den Koschmieder S Berdel WE Serve H Müller-Tidow C The C/EBPδ tumor suppressor is silenced by hypermethylation in acute myeloid leukemia Blood 2007 109 3895 3905 10.1182/blood-2006-08-040147 17234736

[B18] Hackanson B Bennett KL Brena RM Jiang J Claus R Chen SS Blagitko-Dorfs N Maharry K Whitman SP Schmittgen TD Lübbert M Marcucci G Bloomfield CD Plass C Epigenetic modification of CCAAT/enhancer binding protein A expression in acute myeloid leukemia Cancer Res 2008 68 3142 3151 10.1158/0008-5472.CAN-08-0483 18451139

[B19] Jost E do ON Wilop S Herman JG Osieka R Galm O Aberrant DNA methylation of the transcription factor *C/EBPα *in acute myelogenous leukemia Leuk Res 2009 33 443 449 10.1016/j.leukres.2008.07.027 18757096

[B20] Herman JG Graff JR Myöhänen S Nelkin BD Baylin SB Methylation-specific PCR: a novel PCR assay for methylation status of CpG islands Proc Natl Acad Sci USA 1996 93 9821 9826 10.1073/pnas.93.18.9821 8790415 PMC38513

